# Risk stratification and management of arrhythmias in patients with Wolff–Parkinson–White syndrome

**DOI:** 10.1097/MS9.0000000000003068

**Published:** 2025-03-07

**Authors:** Chukwuka Elendu, Festus O. Babarinde, Olusola D. Babatunde, Emmanuel A. Babawale, Jemilah I. Hassan, Victor I. Ikeji, Boluwatife D. Oshin, Adaugo Nwabueze, Jide K. Ngozi-Ibeh, Christopher Chukwu, Osazuwa T. Ighodaro, Mary N. Ikokwu, Oluwatosin A. Akinruli, Consolata I. Uzzi, Onyeanusi C. Sunday, Maryjudith N. Nwachukwu, Chigozie D. Opara, Chinyereugo L. Nwokocha, Chidi A. Udoeze, Peter I. Ogidigba

**Affiliations:** a Department of Internal Medicine, Federal University Teaching Hospital, Owerri, Nigeria; b Department of Internal Medicine, University of Ibadan, Ibadan, Nigeria; c Department of Internal Medicine, Cagayan State University, Tuguegarao, Philippines; d Department of Internal Medicine, Griffith College Dublin, Dublin, Ireland; e Department of Internal Medicine, Nahda University, Beni Suef, Egypt; f Department of Internal Medicine, Ternopil National Medical University, Ternopil, Ukraine; g Department of Internal Medicine, Babcock University, Ilishan-Remo, Ogun State, Nigeria; h Department of Internal Medicine, Kwame Nkrumah University of Science and Technology, Kumasi, Ghana; i Department of Internal Medicine, I. Horbachevsky Ternopil National Medical University, Ternopil, Ukraine; j Department of Internal Medicine, University of Jos, Plateau, Nigeria; k Department of Internal Medicine, Basildon University Hospital, UK; l Department of Internal Medicine, Abia State University, Uturu, Nigeria; m Department of Internal Medicine, Keele University, UK; n Department of Internal Medicine, Bristol Myers Squibb, USA; o Department of Internal Medicine, Nnamdi Azikiwe University, Awka, Nigeria; p Department of Internal Medicine, Imo State University, Owerri, Nigeria; q Department of Internal Medicine, University of Benin, Benin City, Nigeria

**Keywords:** arrhythmias, catheter ablation, management, risk stratification, Wolff–Parkinson–White syndrome

## Abstract

Wolff–Parkinson–White (WPW) syndrome is a cardiac disorder characterized by an accessory pathway known as the bundle of Kent, which bypasses the atrioventricular node and predisposes patients to tachyarrhythmias, including supraventricular tachycardia and atrial fibrillation. The prevalence of WPW syndrome ranges from 0.1% to 0.5% globally, with a higher incidence in males and diagnosis typically occurring between ages 10 and 30. Although many individuals remain asymptomatic, WPW presents a risk of life-threatening arrhythmias, including sudden cardiac death, particularly when atrial fibrillation progresses to ventricular fibrillation (VF). Effective risk stratification is essential for managing WPW syndrome; this involves evaluating symptomatic presentation, accessory pathway properties, and underlying cardiac conditions using clinical, electrocardiographic, and electrophysiological data. Recent advances in risk stratification models enable clinicians to identify better patients at high risk for arrhythmia recurrence or sudden death, informing treatment strategies such as catheter ablation. This study emphasizes the importance of personalized risk assessment in guiding therapeutic decisions, minimizing unnecessary interventions, and improving outcomes. This review’s novel contribution lies in its updated analysis of risk stratification models, which incorporates new research on the genetic and epidemiological factors influencing WPW syndrome. This analysis emphasizes the evolving role of catheter ablation as a first-line treatment for high-risk patients, demonstrating its potential to provide long-term arrhythmia control with minimal complications. Our findings underscore the necessity for ongoing post-ablation surveillance to monitor for recurrence and to optimize patient care, ultimately improving clinical outcomes.

## Introduction and background

Wolff–Parkinson–White (WPW) syndrome is a congenital heart condition characterized by an abnormal electrical conduction pathway known as the bundle of Kent, which bypasses the atrioventricular (AV) node. This abnormality allows preexcitation of the ventricles and can lead to episodes of rapid heart rate (tachycardia) or other arrhythmias, such as paroxysmal supraventricular tachycardia (SVT) and atrial fibrillation (AF)^[1–3]^. Although the syndrome can remain asymptomatic, it is a significant condition due to the potential for life-threatening arrhythmias, including sudden cardiac death (SCD)^[4,5]^. WPW syndrome has a reported prevalence of 0.1%–0.3% in the general population, though this may be underestimated due to undiagnosed asymptomatic cases^[1,6]^. It typically affects 1 in 2000 individuals, with a higher prevalence in males (2:1 male-to-female ratio)^[7,8]^. Genetic factors are thought to play a role in developing WPW, though the precise mechanisms remain unclear^[9]^. The condition can present at any age, often during adolescence or early adulthood. Clinical manifestations range from incidental discovery on an electrocardiogram (ECG) to symptomatic episodes of tachycardia, presenting with palpitations, dizziness, or syncope^[10]^. In severe cases, these arrhythmias can lead to heart failure or sudden cardiac arrest^[11]^. Diagnostic evaluation includes ECG, which reveals a characteristic short PR interval and delta wave, and electrophysiological studies (EPS) to assess the properties of the accessory pathway^[12]^. Management depends on the severity of symptoms and arrhythmia risk. Asymptomatic individuals may require only monitoring, while those with recurrent or severe tachycardia benefit from antiarrhythmic medications or catheter ablation, which is highly effective in interrupting the abnormal pathway^[13–15]^. Recent advancements, such as electroanatomical mapping, have improved the safety and accuracy of ablation procedures. Ongoing research into the genetic and molecular basis of WPW syndrome holds promise for future therapeutic developments^[16,17]^. Long-term follow-up is essential for WPW patients to monitor for new arrhythmias and adjust treatment as needed, ensuring sustained symptom control and mitigating complications^[18–20]^.

**HIGHLIGHTS**

- Identifying high-risk WPW patients for sudden cardiac events.
- Effective strategies for arrhythmia management and prevention.

## Data collection

The review was conducted through a systematic search of peer-reviewed literature published up to August 2024. Key databases, including PubMed, Embase, and the Cochrane Library, were utilized to identify relevant articles. Search terms included “Wolff-Parkinson-White syndrome,” “risk stratification,” “arrhythmias,” “management strategies,” “catheter ablation,” and “electrophysiological studies.” The search was limited to English-language articles published in reputable, peer-reviewed journals. Only peer-reviewed research articles, systematic reviews, and clinical guidelines on WPW syndrome were considered. Selected studies provided information on risk stratification models, management strategies, and patient outcomes related to WPW syndrome. This included data on clinical characteristics, parameters of risk stratification models, and the efficacy of management approaches such as pharmacological treatments and catheter ablation. Studies, including electrophysiological findings and treatment outcomes, were prioritized to ensure a thorough review of current practices. Articles that did not specifically address WPW syndrome or focused on unrelated arrhythmias were excluded. Non-peer-reviewed sources, editorials, opinion pieces, and studies lacking detail or relevance to the review’s objectives were also omitted.

Data extraction involved systematically collecting information from the selected studies on various aspects of WPW syndrome management. Key data points included patient demographics, clinical features, and parameters of risk stratification models. Detailed information on management strategies was gathered, focusing on pharmacological treatments, catheter ablation techniques, and their outcomes. The effectiveness of these interventions was assessed based on reported patient outcomes and long-term follow-up data. The extracted data were organized to identify common themes and insights across different studies, evaluating the applicability and efficacy of risk stratification models and management strategies in clinical practice. Integrating findings involved synthesizing data from multiple sources to overview the current knowledge of WPW syndrome. This process required evaluating and comparing the effectiveness of various risk stratification models and management strategies. The findings were organized to highlight common approaches and differences in risk assessment and treatment across studies. By integrating data on patient outcomes, treatment efficacy, and risk stratification parameters, the review aimed to provide a clear understanding of WPW syndrome management. Gaps in the existing literature were also identified, and recommendations for future research were proposed to improve clinical practices.

## Prevalence, etiology, and pathophysiology

WPW syndrome is observed in approximately 0.1%–0.3% of the general population, though prevalence may reach as high as 0.5% in certain subgroups^[1,2]^. This variability reflects differences in diagnostic sensitivity and population genetics. Notably, WPW syndrome is diagnosed more frequently in males than females, with a reported male-to-female ratio of 2:1^[3]^. The syndrome commonly presents during adolescence or early adulthood, between the ages of 10 and 30, often due to increased diagnostic detection of arrhythmias during these years, either symptomatically or incidentally, on ECGs^[1,21,22]^. Although WPW syndrome is congenital, symptoms may not manifest immediately and can emerge later in life. Genetics plays a significant role in WPW syndrome, as family studies indicate a higher prevalence among first-degree relatives of affected individuals^[4]^. Specific genetic mutations, such as those in the PRKAG2 gene, have been linked to familial cases of WPW, particularly when associated with glycogen storage diseases and hypertrophic cardiomyopathy (HCM).

Furthermore, genome-wide association studies have identified potential loci that influence the formation of accessory pathways, although the full genetic basis of WPW remains incompletely understood^[5]^. The prevalence and clinical presentation of WPW syndrome also show variation across different geographic and demographic groups. The comparison of WPW prevalence, incidence, gender distribution, age of onset, and the risk of SCD across different regions and countries are displayed in Table 1. Notably, studies suggest that individuals of East Asian descent may have a higher prevalence of WPW syndrome, while African Americans may exhibit a lower prevalence^[8]^. These disparities may be influenced by genetic factors, healthcare access, and diagnostic utilization rates, complicating the epidemiological landscape. The etiology of WPW syndrome is multifactorial. Congenital factors are pivotal, with accessory pathways arising from developmental abnormalities during embryogenesis. Disruptions in the formation of the normal conduction system lead to the creation of abnormal myocardial connections between the atria and ventricles. Genetic mutations have been implicated in this abnormal development, particularly those affecting ion channels and cardiac structural proteins. For instance, mutations in the PRKAG2 gene, encoding the γ2 subunit of AMP-activated protein kinase, are associated with familial WPW and HCM^[23]^.

**Table 1:** Geographic variation in the epidemiology of Wolff–Parkinson–White syndrome

Region/country	Prevalence (%)	Incidence (per 100 000/year)	Gender ratio (M)	Typical age of onset	Risk of sudden cardiac death (%)	Common genetic factors	Healthcare access	Diagnostic rate
United States	0.1–0.3	3–4	2:1	10–30 years	0.1–0.2	PRKAG2 mutation	High	High
Japan	0.2–0.4	2–3	2:1	Adolescence to early adulthood	0.1	Not well defined	Moderate	Moderate
India	0.1–0.3	2–4	3:1	Childhood to adolescence	0.1–0.2	Familial cases reported	Variable	Low to moderate
China	0.3–0.5	5–6	1.5:1	Early adulthood	0.15–0.2	GWAS-linked loci	Moderate	High
United Kingdom	0.1–0.2	1–2	2:1	Adolescence to early adulthood	0.1	PRKAG2 mutation	High	High
Brazil	0.1–0.3	3–4	2.5:1	Childhood to adolescence	0.1–0.15	Familial cases reported	Moderate	Moderate
South Africa	0.05–0.1	1–2	2:1	Adolescence	0.05–0.1	Limited data	Low to moderate	Low
Nigeria	0.05–0.2	2–3	1.8:1	Adolescence to early adulthood	0.05–0.1	Limited data	Low	Low

This table displays the epidemiological differences of WPW syndrome across various regions and countries, highlighting how factors such as prevalence, incidence, gender ratio, age of onset, risk of sudden cardiac death, genetic predisposition, healthcare access, and diagnostic rates can vary geographically. For instance, the prevalence and incidence rates are generally higher in East Asian countries like Japan and China compared to Western countries such as the United States and the United Kingdom. This could be due to genetic factors, as suggested by specific genetic mutations and loci in these populations, as well as differences in healthcare access and diagnostic practices. The gender ratio also shows variability, with some regions reporting a higher male predominance. The typical age of onset for WPW syndrome tends to cluster around adolescence to early adulthood across most regions, reflecting the period when arrhythmias are more likely to be detected either symptomatically or through routine screenings. The risk of sudden cardiac death varies, often correlating with the severity of arrhythmias and the availability of advanced cardiac care facilities. Regions with higher healthcare access and diagnostic rates, such as the United States and the United Kingdom, tend to have more robust data collection and reporting systems, leading to more accurate prevalence and incidence estimates. In contrast, countries with lower healthcare access, such as South Africa and Nigeria, may have underreported cases due to limited diagnostic resources and healthcare infrastructure. [Authors Creations.]

Additionally, transcription factors such as NKX2-5 and GATA4 and alterations in pathways like Wnt/β-catenin have been implicated in the developmental processes that result in accessory pathways^[24–26]^. While genetic predispositions are central, environmental factors may also influence the development of WPW syndrome. Drugs that affect ion channels prolong the QT interval or cause electrolyte disturbances have been linked to acquired forms of WPW syndrome. Moreover, prenatal exposure to alcohol or certain medications could disrupt cardiac development, predisposing individuals to congenital heart defects associated with WPW syndrome^[27]^. At the core of WPW syndrome is an accessory pathway known as the bundle of Kent, which allows electrical impulses to bypass the AV node, resulting in rapid conduction between the atria and ventricles. This abnormal conduction leads to a shortened PR interval, a widened QRS complex, and the characteristic delta wave on an ECG^[17]^. The accessory pathway predisposes individuals to tachyarrhythmias, including atrioventricular reentrant tachycardia (AVRT) and AF, which can lead to life-threatening complications like VF^[6]^. The accessory pathway’s location and properties, such as its refractory period and conduction velocity, are critical in determining the risk and type of arrhythmias that may develop. Pathways with short refractory periods, or those near the AV node, are more likely to facilitate rapid conduction and predispose individuals to arrhythmias.

In contrast, others may have slower conduction and lower arrhythmogenic potential^[19]^. Additionally, variations in accessory pathway characteristics, such as decremental or supernormal conduction, significantly influence the effectiveness of antiarrhythmic treatments and interventions like catheter ablation^[28]^. From a public health perspective, WPW syndrome imposes clinical and economic burdens. Management often requires regular monitoring and potentially invasive interventions, such as catheter ablation, contributing to healthcare costs. In addition, the risk of recurrent arrhythmias and SCD poses psychological and social implications for affected individuals, particularly those experiencing frequent arrhythmias or anxiety over severe complications^[29–31]^.

## Clinical presentation

Palpitations are the most common symptom in individuals with WPW syndrome, often described as rapid, pounding, or irregular heartbeats. They may occur spontaneously or be triggered by physical exertion, emotional stress, or stimulant use. Patients frequently report sensations of “skipped” beats or “fluttering” in the chest, which can be accompanied by anxiety or discomfort. The frequency and duration of palpitations can vary significantly. Dizziness and lightheadedness are also prevalent and may result from hemodynamic compromise during rapid or irregular heart rhythm episodes. Patients may experience weakness, fatigue, or pre-syncope, impairing daily activities and exercise. In severe cases, syncope (loss of consciousness) can occur, often due to significant reductions in cardiac output or cerebral perfusion from arrhythmias like AVRT or AF.

Syncope can happen suddenly, leading to falls or injuries. Chest pain is less common but can occur, typically described as pressure or tightness. This pain may arise spontaneously or be triggered by exertion or stress and could be linked to increased myocardial oxygen demand or reduced coronary perfusion during arrhythmias. Shortness of breath (dyspnea) may occur, especially during episodes that compromise cardiac output. Individuals with underlying heart disease may experience exacerbated symptoms, necessitating prompt evaluation to prevent decompensation. Fatigue and malaise are nonspecific symptoms that may arise during arrhythmia-related hemodynamic instability. They can be worsened by physical exertion or stress and interfere with daily life. Chronic fatigue may also relate to comorbidities like anxiety or depression. Psychological distress, including anxiety and depression, is common, particularly among those with recurrent arrhythmias or syncope. The unpredictability of these events can lead to significant morbidity, including fear of sudden death and social isolation. However, the clinical presentation of WPW syndrome varies widely among individuals, influenced by age, sex, comorbidities, and the presence of underlying heart disease. Some individuals remain asymptomatic, with WPW detected incidentally during routine evaluations. Even asymptomatic patients may be at risk for arrhythmias and SCD, necessitating periodic monitoring and risk stratification.

## Clinical vignette

This patient’s history is based on an original case observed and managed by the authors at our clinic. A 34-year-old female presented with a complex constellation of symptoms that ultimately led to the diagnosis and management of WPW syndrome. Her case underscores the critical importance of meticulous assessment and tailored management in patients with this condition. The patient initially presented with a history of recurrent palpitations and episodes of lightheadedness over the past 18 months. She described the palpitations as sudden onset, lasting for several minutes, and often associated with dizziness and shortness of breath. Her episodes were infrequent but had become progressively more intense and distressing, prompting her to seek medical attention. Upon further inquiry, it was revealed that the patient had no significant past medical history. She was otherwise healthy and did not have any notable family history of cardiovascular disease. She was a nonsmoker with no history of excessive alcohol consumption or drug use. Her physical examination was largely unremarkable, except for a regular pulse and blood pressure within normal limits. However, an ECG conducted during an episode of palpitations showed characteristic findings of WPW syndrome, including a short PR interval and a delta wave, indicative of preexcitation. Given the clinical presentation and ECG findings, a referral to a cardiologist was made for further evaluation. The cardiologist, noting the presence of symptoms consistent with WPW syndrome, recommended an ambulatory Holter monitor to assess the frequency and duration of arrhythmias. The Holter monitor captured several episodes of SVT, reinforcing the suspicion of WPW syndrome and demonstrating the need for intervention. The patient was subsequently referred for an EPS. The EPS revealed the presence of a concealed accessory pathway, which was responsible for the recurrent episodes of SVT. The study also highlighted that the accessory pathway was located on the left side of the heart, a finding that guided the subsequent management plan. The patient was advised to undergo catheter ablation, which is considered the treatment of choice for symptomatic WPW syndrome, particularly in the presence of a significant accessory pathway. The catheter ablation procedure used radiofrequency energy to destroy the aberrant conduction pathway. The procedure was successful, with no immediate complications, and post-procedure ECG showed no evidence of the accessory pathway or abnormal conduction. The patient was monitored in the recovery unit and discharged with instructions to follow up in the clinic for ongoing assessment. Following the ablation, the patient reported significantly reducing the frequency and severity of her arrhythmia episodes. Her follow-up visits included regular clinical evaluations and repeated ECGs to ensure that the arrhythmias had not recurred. During these visits, the patient was encouraged to use a wearable ECG monitor to track her heart rhythms and provide real-time data to her healthcare provider. This technology proved invaluable, allowing continuous monitoring and immediate feedback, helping reassure the patient and the medical team. Over the subsequent months, the patient remained asymptomatic, and follow-up ECGs consistently showed normal sinus rhythm with no evidence of preexcitation. The wearable device confirmed the absence of arrhythmias and reassured the patient. Regular follow-up and monitoring were emphasized, including maintaining a healthy lifestyle and adhering to prescribed medications as necessary. The case of this patient highlights the intricate nature of diagnosing and managing WPW syndrome. It illustrates the effectiveness of catheter ablation as a treatment for symptomatic WPW syndrome and the role of advanced diagnostic tools, such as EPS and wearable technology, in guiding and monitoring treatment. The patient’s successful outcome underscores the importance of individualized care and ongoing follow-up in managing complex arrhythmias. Upon entering the examination room, the patient appeared alert and well-nourished, with no signs of acute distress. She was dressed appropriately for the weather and was calm and cooperative. The examination began with a thorough assessment of her general appearance and vital signs, which provided essential baseline information. The patient’s vital signs were recorded: her blood pressure was 118/76 mmHg, her heart rate was 78 beats per minute, her respiratory rate was 16 breaths per minute, and her temperature was 98.6°F (37°C). These values were within normal limits, and there were no indications of fever, hypotension, or tachycardia at rest. Her body mass index was 22.5 kg/m^2^, indicating a normal weight for her height and age. Inspection of the patient’s skin revealed no rashes, lesions, or cyanosis. The skin was warm and dry, with no signs of pallor or jaundice. Her extremities were well-perfused, with normal capillary refill time and no evidence of edema. The patient’s jugular venous pressure (JVP) was assessed at a 45° angle. The JVP was within normal limits, with no signs of elevated venous pressure that could suggest right heart failure or fluid overload. A thorough cardiovascular examination was performed, beginning with palpation of the precordium. The chest was inspected for any visible pulsations, and palpation revealed a normal point of maximal impulse located in the fifth intercostal space at the midclavicular line. No abnormal thrills or heaves were detected. The heart sounds were auscultated using a stethoscope placed at the four traditional areas: the aortic, pulmonic, tricuspid, and mitral regions. The heart sounds were regular and normal in frequency and intensity. No additional heart sounds, such as S3 or S4, were heard, and no abnormal murmurs or extra sounds were detected. The auscultation of the heart revealed a normal S1 and S2, with no evidence of split sounds or abnormal murmurs. Palpation of the peripheral pulses was performed to assess for any abnormalities in the arterial system. The radial pulses were found to be equal and symmetric bilaterally, with a normal rate and rhythm. The femoral pulses were palpable and symmetric, as were the popliteal, dorsalis pedis, and posterior tibial pulses. The quality of the pulses was strong and regular, with no signs of diminished or absent pulses that could suggest peripheral arterial disease or other vascular issues. A thorough respiratory examination was conducted next. The patient’s chest was inspected for signs of respiratory distress or abnormal respiratory patterns. The patient did not use accessory muscles or signs of labored breathing. Palpation of the chest wall revealed no tenderness or abnormal vibrations. Percussion of the lung fields was performed to assess for any abnormalities in lung density or the presence of fluid or consolidation. Normal resonance was noted over all lung fields, with no dullness or hyperresonance that could indicate pathological changes. Auscultation of the lung fields revealed clear breath sounds bilaterally, with no added sounds such as wheezes, crackles, or rhonchi. The breath sounds were equal and symmetrical, indicating normal airflow through the lungs. The patient demonstrated a normal respiratory pattern with no signs of wheezing or crackles that could suggest underlying respiratory pathology. The abdominal examination was conducted to assess for any signs of abdominal or systemic disease. The abdomen was inspected for visible abnormalities, such as distension or asymmetry. Palpation of the abdomen revealed no tenderness, masses, or organomegaly. The liver and spleen were not palpable below the costal margins, and there were no signs of ascites. Percussion of the abdomen produced normal tympanic sounds, and bowel sounds were auscultated as normal, indicating healthy gastrointestinal function. Finally, a brief neurological examination was performed to ensure no signs of neurological deficits could explain the patient’s episodes of dizziness. The patient was alert and oriented to person, place, and time. Cranial nerves II through XII were intact, with normal visual acuity, pupillary reactions, and extraocular movements. Motor and sensory examinations revealed normal strength and sensation in all extremities, with no signs of weakness, numbness, or abnormal reflexes.

## Risk stratification

Risk stratification in patients with WPW syndrome is essential for determining the likelihood of adverse outcomes and guiding appropriate management strategies. WPW syndrome, characterized by the presence of an accessory conduction pathway leading to episodes of SVT or AF, necessitates accurate risk assessment to prevent serious complications such as SCD. Various risk stratification models and scoring systems have been developed to assess the severity of WPW syndrome, using clinical, electrocardiographic, and electrophysiological parameters to stratify risk and guide treatment decisions.

### Diagnostic modalities and noninvasive tools in WPW syndrome

The adenosine triphosphate (ATP) test is a valuable and widely utilized noninvasive diagnostic tool for assessing conduction abnormalities. In WPW syndrome, the accessory pathway enables rapid electrical conduction that may lead to reentrant tachycardias, with potential implications for morbidity and, in rare cases, SCD. This pathway guides treatment decisions, including pharmacological therapy, catheter ablation, or observation. The ATP test works by exploiting the physiological effects of adenosine on the AV node^[5–7]^. Adenosine, a purinergic receptor agonist, acts on the A1 adenosine receptors in the AV node to induce transient AV nodal block, thereby preventing conduction through the AV node and allowing any bypass conduction through accessory pathways to manifest more prominently. This property makes the ATP test a cornerstone for diagnosing and risk-stratifying patients with WPW syndrome. One of the major advantages of the ATP test is its noninvasive nature. Unlike invasive EPS, which require catheter placement and fluoroscopic imaging, the ATP test can be performed as an outpatient procedure. This simplicity is particularly advantageous for initial risk stratification or in cases where EPS is not immediately feasible. For example, the ATP test is often used in asymptomatic patients with ECG findings suggestive of WPW syndrome to determine the presence of high-risk conduction properties^[8,9]^. In these scenarios, the ATP test can help identify patients who may benefit from further invasive evaluation or definitive treatment with catheter ablation. The ATP test’s diagnostic utility extends beyond WPW syndrome. It is also instrumental in differentiating WPW from other conditions that can mimic preexcitation, such as atrioventricular nodal reentrant tachycardia (AVNRT) or fasciculoventricular pathways (FVP). In AVNRT, the reentrant circuit is confined to the AV node, and the absence of an accessory pathway can be confirmed by the transient AV nodal block induced by ATP^[10,11]^. Similarly, in cases of FVP, the ATP test reveals that preexcitation persists despite AV nodal blockade, confirming the accessory pathway’s conduction properties. These distinctions are critical for ensuring accurate diagnosis and avoiding unnecessary interventions, as FVP and AVNRT are typically managed differently from WPW syndrome. The ATP test has also been studied extensively in pediatric populations, where noninvasive diagnostic strategies are crucial due to the potential risks associated with invasive procedures. Research has demonstrated that ATP testing is safe and effective in children with suspected WPW syndrome, providing diagnostic clarity without the need for sedation or invasive EPS^[12–14]^. Moreover, in pediatric and adult populations alike, the ATP test is a useful adjunct to ECG and Holter monitoring for capturing transient arrhythmias and confirming the diagnosis of preexcitation syndromes. Although the ATP test is generally well-tolerated, it is not without limitations. Some patients experience transient side effects, such as chest discomfort, flushing, or dyspnea, due to the systemic vasodilatory effects of adenosine. However, these effects are short-lived and self-limiting, making the ATP test a safe option for most patients. Additionally, the test’s sensitivity and specificity depend on appropriate patient selection and interpretation by experienced clinicians. In rare cases, false negatives may occur if the accessory pathway’s conduction properties are intermittent or if adenosine fails to produce adequate AV nodal blockade^[15–17]^. The ATP test is often combined with other diagnostic modalities, such as ECG, echocardiography, or EPS, to evaluate and mitigate these limitations comprehensively. The ATP test has significantly advanced, enhancing its diagnostic precision and clinical utility. For instance, developing protocols for standardized adenosine dosing and administration has minimized variability in test results and improved reproducibility. Additionally, advances in ECG technology, including high-resolution mapping and automated analysis, have complemented the ATP test by enabling more detailed characterization of arrhythmogenic substrates and conduction pathways. These innovations have expanded the ATP test’s applications, making it a cornerstone in the diagnostic algorithm for WPW syndrome and related conditions^[18,19]^. The ATP test is pivotal in clinical practice in managing patients with WPW syndrome. The ATP test provides critical information for risk stratification and therapeutic decision-making for symptomatic patients presenting with palpitations, syncope, or documented arrhythmias. Specifically, the test helps determine whether the accessory pathway is capable of conducting at high rates, which is a key risk factor for life-threatening arrhythmias such as AF or VF. Based on the ATP test results, clinicians can tailor treatment plans to the individual patient’s risk profile, ranging from conservative monitoring to aggressive interventions such as catheter ablation. The ATP test is equally valuable in asymptomatic patients with incidental findings of preexcitation on ECG. These patients often pose a diagnostic and therapeutic dilemma, as the natural history of WPW syndrome is highly variable, and not all individuals with preexcitation are at risk for adverse outcomes^[20,28]^. The ATP test provides an objective and noninvasive means of identifying high-risk conduction properties, facilitating early intervention in patients who might otherwise go unrecognized. This proactive approach has been shown to reduce the incidence of arrhythmia-related complications and improve long-term outcomes in WPW syndrome. The ATP test also has important implications for population health and resource allocation. By providing a cost-effective and noninvasive diagnostic option, the ATP test reduces the need for more expensive and invasive procedures, such as EPS or advanced imaging studies. This is particularly relevant in resource-limited settings, where access to specialized electrophysiological services may be constrained. The widespread availability and simplicity of the ATP test make it an ideal tool for expanding access to arrhythmia care and improving diagnostic accuracy in diverse healthcare settings^[32,33]^. Despite numerous advantages, the ATP test is not a standalone solution for diagnosing WPW syndrome. It should be integrated into a comprehensive diagnostic strategy incorporating clinical history, physical examination, and complementary diagnostic modalities. For example, the ATP test is often combined with exercise stress testing or pharmacological challenge to assess the accessory pathway’s behavior under varying physiological conditions. Additionally, advancements in genetic testing and molecular biology have provided new insights into the pathophysiology of WPW syndrome, offering opportunities to further refine diagnostic and therapeutic approaches^[1,21]^.

One of the primary noninvasive tools used in WPW syndrome is electrocardiography (ECG). The ECG is fundamental in diagnosing WPW syndrome by identifying the characteristic delta wave, which represents preexcitation of the ventricles due to an accessory pathway bypassing the AV node. Besides diagnosis, ECG parameters can also provide prognostic information^[34]^. For instance, a critical marker is the shortest pre-excited R-R interval during atrial fibrillation (SPERRI) on a standard ECG. A SPERRI of less than 250 milliseconds is associated with a higher risk of rapid ventricular response and potentially life-threatening arrhythmias, such as VF. The measurement of SPERRI is particularly useful in asymptomatic patients, as it helps identify those who may be at risk of SCD despite the absence of symptoms^[20]^. The ambulatory 24-hour Holter monitoring is another valuable noninvasive tool for risk stratification in WPW syndrome. This extended ECG monitoring detects intermittent arrhythmias that may not be captured during a standard ECG. It provides information on the frequency and duration of arrhythmic episodes, symptomatic or asymptomatic episodes, and heart rate variability. Holter monitoring can identify high-risk features such as frequent premature ventricular contractions, sustained episodes of SVT, or periods of rapid ventricular response during AF. These findings can indicate an increased risk of adverse events and guide further diagnostic and therapeutic interventions^[28]^. Exercise stress testing is a noninvasive tool used to evaluate the behavior of the accessory pathway during physical exertion. This test is particularly useful in young, asymptomatic patients with WPW syndrome, as it can unmask high-risk features that may not be apparent at rest. During exercise, sympathetic stimulation can enhance conduction through the accessory pathway, potentially inducing arrhythmias^[6]^. A normal response is characterized by the sudden loss of preexcitation with increased heart rate. This indicates that the accessory pathway cannot conduct at higher rates, which is generally associated with a lower risk of SCD. Conversely, persistence or enhancement of preexcitation during exercise may indicate a more malignant accessory pathway capable of rapid conduction, thereby increasing the risk of SCD. The predictive value of exercise stress testing lies in its ability to provoke arrhythmias and assess the functional properties of the accessory pathway under stress conditions^[32]^. Echocardiography is another important noninvasive modality in the risk stratification of WPW syndrome, particularly for assessing the structural and functional status of the heart. While WPW syndrome is primarily an electrical disorder, the presence of structural heart disease can significantly alter the risk profile. Echocardiography can identify coexisting congenital heart defects, such as Ebstein’s anomaly, HCM, or dilated cardiomyopathy, which are associated with a higher risk of arrhythmic events^[9]^. In addition, echocardiography can assess left ventricular function, ventricular size, and the presence of valvular abnormalities, which may influence the choice of treatment and prognosis. For instance, patients with reduced left ventricular ejection fraction or significant left ventricular hypertrophy may require more aggressive management and closer follow-up^[10]^. Cardiac magnetic resonance imaging (MRI) and computed tomography (CT) are advanced imaging modalities that offer detailed anatomical and functional information. Although not routinely used for all WPW patients, they can be valuable in complex cases where precise localization of the accessory pathway or assessment of structural abnormalities is necessary. Cardiac MRI, in particular, provides superior soft tissue contrast and can evaluate myocardial fibrosis, which may contribute to arrhythmogenesis^[11]^. Additionally, MRI and CT can delineate the anatomy of the accessory pathway and its relationship to the surrounding cardiac structures, aiding in pre-procedural planning for catheter ablation. These modalities also have a role in identifying and characterizing myocardial scars or other structural abnormalities that may not be apparent on echocardiography. Genetic testing is an emerging tool in the risk stratification of WPW syndrome, particularly in patients with a family history of arrhythmias or SCD^[12]^. Genetic testing can identify mutations in genes associated with arrhythmic syndromes, such as those affecting ion channels or structural proteins. For example, mutations in the PRKAG2 gene have been linked to a syndrome characterized by ventricular preexcitation, HCM, and glycogen storage disease. Identifying such mutations can provide insight into the pathophysiological mechanisms underlying the arrhythmia, guide management decisions, and inform family screening^[13]^. While genetic testing is not yet routinely recommended for all patients with WPW syndrome, it holds promise for personalized medicine approaches and better risk stratification. Another noninvasive tool that recently gained attention is signal-averaged ECG (SAECG). SAECG enhances the detection of late potentials, which are low-amplitude signals occurring after the QRS complex^[14]^. The presence of late potentials is associated with an increased risk of ventricular arrhythmias and SCD. In the context of WPW syndrome, SAECG may provide additional prognostic information, particularly in patients with structural heart disease or unexplained syncope. The utility of SAECG in WPW syndrome is still under investigation, and further studies are needed to establish its role in clinical practice^[15]^. Electrophysiological testing (EPT) is often considered the gold standard for risk stratification in WPW syndrome. However, it is an invasive procedure, and noninvasive methods are preferred for initial risk assessment. EPT involves the insertion of catheters into the heart to directly measure electrical activity and assess the properties of the accessory pathway. It can induce arrhythmias, evaluate the shortest pre-excited R-R interval during AF, and determine the accessory pathways and AV node’s refractory periods. While EPT provides definitive information about the arrhythmic risk, it is associated with procedural risks and discomfort for the patient^[16]^. Noninvasive tools can often provide sufficient information to guide management decisions without invasive testing. Wearable technology, such as smartwatches and portable ECG devices, has also emerged as a noninvasive method for monitoring and risk stratification in WPW syndrome^[17]^. These devices can continuously monitor heart rate and rhythm, detect arrhythmias in real-time, and alert patients and healthcare providers to abnormal findings. Wearable technology can be particularly useful for capturing transient or asymptomatic arrhythmias that might not be detected during routine clinical visits. The integration of wearable devices into patient care has the potential to enhance arrhythmia detection, improve patient engagement, and facilitate timely interventions^[18]^. Noninvasive risk stratification tools are not without limitations. The accuracy of these tools can be affected by technical factors, such as signal quality, and patient factors, such as body habitus or comorbidities. Interpreting noninvasive tests requires expertise and experience, as false positives or negatives can lead to inappropriate management decisions^[19]^. The sensitivity and specificity of each tool can vary, and combining multiple modalities may improve diagnostic accuracy and risk assessment. For example, combining ECG parameters with imaging findings or Holter monitoring results may provide a more comprehensive risk profile than using any single modality alone^[20]^. The evaluation of risk stratification, as summarized in Table 2, informs management decisions for patients with WPW syndrome. Factors such as symptom duration, arrhythmia frequency, and the presence of structural heart disease are critical in guiding treatment options, including the potential for catheter ablation.

**Table 2:** Risk stratification models and scoring systems for Wolff–Parkinson–White syndrome

Model/score system	Key parameters	Strengths	Limitations	Risk stratification	Guiding interventions	Evidence base	Application context
Risk stratification model	Accessory pathway location, conduction properties	Focuses on pathway characteristics, high predictive value for severe cases	It does not account for comorbid conditions, limited to electrophysiological data	High-risk vs. Low-risk	Catheter ablation, close monitoring	Strong evidence from electrophysiological studies^[13]^	Patients with symptomatic WPW syndrome
Comprehensive model	Syncope history, atrial fibrillation, EPS results	Incorporates clinical history and EPS findings, comprehensive	It may be less applicable in asymptomatic patients, with less focus on new advancements	High-risk vs. moderate-risk	Aggressive treatment, ablation, lifestyle modification	Supported by clinical trials and cohort studies^[14]^	Patients with recurrent arrhythmias
Risk stratification score	Duration of symptoms, frequency of arrhythmias, structural heart disease	Provides a numerical risk score, easy to use in clinical practice	May oversimplify risk assessment, variability in scoring criteria	Risk categories (e.g. low, moderate, high)	Tailored treatment plans based on score	Developed from diverse clinical data^[15]^	Broad clinical application, including general and specialized settings

Each model and scoring system offers a unique perspective on risk stratification, and their application depends on specific clinical contexts and patient characteristics. The Risk Stratification Model is particularly useful for patients with clear electrophysiological findings, while the Comprehensive Model provides a more integrated approach by including clinical history and EPS results. The Risk Stratification Score is valuable for its ease of use and numerical approach, though it may only capture some nuances of individual patient risk. The strengths of these models include their ability to guide clinical decision-making and stratify patients based on their risk of serious arrhythmias. They help identify patients most likely to benefit from catheter ablation or more intensive monitoring interventions. However, limitations include reliance on specific parameters that may not be universally applicable and potential variability in risk assessment based on different clinical contexts. In clinical practice, the choice of risk stratification model or scoring system should be guided by the individual patient’s presentation, electrophysiological and clinical data availability, and the overall clinical context. Integrating multiple sources of information and a comprehensive approach can help achieve the most accurate risk assessment and guide appropriate management strategies for patients with WPW syndrome.

## Differential diagnosis of fasciculoventricular pathway

Both FVP and WPW can clinically present asymptomatic findings during routine ECG or incidental findings in patients being evaluated for unrelated complaints. However, WPW syndrome is frequently associated with recurrent episodes of paroxysmal supraventricular tachycardia (PSVT) or AF. These arrhythmias result from the ability of the accessory pathway in WPW to participate in reentrant circuits, often leading to rapid ventricular rates during AF, a potentially life-threatening condition. In contrast, FVP is considered non-arrhythmogenic and is not associated with any symptoms of palpitations, syncope, or sudden cardiac arrest. Its primary significance lies in the potential for misdiagnosis and overtreatment^[1–3]^. Therefore, a thorough clinical evaluation to identify symptoms of arrhythmias or a history of tachycardia is a critical first step in the differential diagnosis. Electrocardiographic features provide key distinguishing characteristics between FVP and WPW syndrome. Both conditions can exhibit preexcitation, manifesting as a delta wave and a short PR interval. However, the QRS complex in FVP is typically narrow because the conduction through the fasciculoventricular pathway directly activates the Purkinje system without significant ventricular myocardial activation^[4–6]^. This contrasts with WPW syndrome, where the QRS complex is often widened due to myocardial preexcitation through the bundle of Kent. In FVP, delta waves are usually subtle and consistent with limited preexcitation. Additionally, there is no change in the QRS morphology during exercise or pharmacologic maneuvers like adenosine administration. In WPW syndrome, the degree of preexcitation may vary with changes in autonomic tone, often producing dynamic ECG changes. Therefore, the narrow QRS complex and stable ECG morphology in FVP are distinguishing features. Electrophysiologic study (EPS) remains the gold standard for differentiating FVP from WPW syndrome. In WPW, the accessory pathway allows retrograde and antegrade conduction, contributing to reentrant arrhythmias^[7–9]^. During EPS, programmed electrical stimulation often reveals conduction through the accessory pathway, and induction of arrhythmias is common. Furthermore, atrial pacing in WPW can demonstrate preexcitation with progressive shortening of the PR interval as conduction occurs through the bundle of Kent. Conversely, in FVP, the accessory pathway does not contribute to arrhythmogenic circuits, and there is no inducible arrhythmia during EPS. Importantly, pacing maneuvers in FVP reveal normal AV nodal conduction without evidence of decremental conduction through the accessory pathway. These findings highlight the benign nature of FVP and underscore the importance of electrophysiologic assessment in the diagnostic process. From a pathophysiologic standpoint, WPW syndrome poses significant risks due to the accessory pathway’s potential to facilitate rapid antegrade conduction during AF, leading to VF^[10,11]^. Consequently, patients with WPW often require risk stratification and, in symptomatic cases, catheter ablation of the accessory pathway. In contrast, FVP does not pose such risks. Given its non-arrhythmogenic nature, treatment is unnecessary, and patients can be reassured. This distinction has important implications for clinical management, emphasizing the need for precise diagnosis to avoid unnecessary procedures. The differential diagnosis of FVP and WPW includes noninvasive diagnostic modalities such as pharmacologic testing. Adenosine or ATP administration can transiently block AV nodal conduction, revealing underlying preexcitation patterns^[12–14]^. In WPW syndrome, AV node blockade unmasks conduction through the accessory pathway, often resulting in marked preexcitation and widening of the QRS complex. In contrast, patients with FVP show no significant changes in ECG morphology during pharmacologic testing, as conduction through the fasciculoventricular pathway bypasses the AV node without contributing to preexcitation. This characteristic makes adenosine testing a useful noninvasive tool in differential diagnosis. AVNRT is another consideration when evaluating a patient with suspected FVP. AVNRT involves reentry within the AV node and presents with regular, narrow QRS complex tachycardias that are sometimes confused with those arising from accessory pathways. Unlike FVP, AVNRT lacks any evidence of accessory pathway conduction on resting ECG^[15,16]^. Additionally, intracardiac EPS demonstrate dual AV nodal physiology and reentrant circuit localization in the AV node for AVNRT, which is not observed in FVP^[4,5]^. Mahaim fibers, another rare type of accessory pathway, often complicate the differential diagnosis. Mahaim fibers originate from the AV node, His bundle, or proximal fascicles and insert into the ventricular myocardium, producing a wide-complex tachycardia with left bundle branch block morphology during tachycardia. Unlike FVP, Mahaim fibers exhibit decremental conduction properties associated with tachyarrhythmias, such as antidromic AVRT^[6]^. The distinction between FVP and Mahaim fibers is crucial due to the therapeutic implications, as Mahaim fibers often necessitate ablation therapy to manage associated arrhythmias. Idiopathic ventricular tachycardia (IVT) must also be considered. IVT originates within the ventricles and can mimic accessory pathway-mediated arrhythmias. IVT commonly exhibits monomorphic wide-complex tachycardia without evidence of preexcitation on baseline ECG. EPS reveals ventricular ectopic foci, often localized to the right ventricular outflow tract or other specific sites, distinct from the fasciculoventricular connections seen in FVP^[7]^. Congenital long QT syndrome (LQTS) and Brugada syndrome also pose diagnostic challenges. LQTS is characterized by a prolonged QT interval and susceptibility to torsades de pointes. At the same time, Brugada syndrome demonstrates coved-type ST-segment elevations in the right precordial leads with a predisposition to polymorphic ventricular tachycardia and SCD. These conditions lack the accessory pathway involvement in FVP but may coexist with other electrophysiological abnormalities that confound diagnosis^[8,9]^. In patients with structural heart disease, bundle branch reentry ventricular tachycardia (BBRVT) may simulate FVP. BBRVT involves reentry within the His-Purkinje system, producing monomorphic wide-complex tachycardia. Unlike FVP, BBRVT requires the presence of underlying His-Purkinje system conduction abnormalities, which are identifiable through EPS. Moreover, BBRVT often occurs in patients with significant cardiomyopathies, which are absent in cases of isolated FVP^[10]^. Fibrosis or scarring from prior cardiac surgeries or myocardial infarction can also create atypical conduction pathways that mimic the electrophysiological behavior of FVP. Post-surgical arrhythmias, such as those following the repair of congenital heart defects, often involve macro-re-entrant circuits or scar-related focal tachycardias. These arrhythmias are distinguishable from FVP through their absence of direct fascicular connections and their dependence on the presence of scar tissue^[11]^. Additionally, certain drug-induced changes in cardiac conduction, including those caused by antiarrhythmic drugs, may mimic the benign conduction seen in FVP. Sodium channel blockers, such as flecainide, can alter conduction velocities, sometimes producing ECG patterns that resemble accessory pathway involvement. However, a careful review of clinical history and drug use typically reveals the reversible nature of these changes and distinguishes them from congenital conditions like FVP^[12]^. Psychogenic pseudosyncope and non-cardiac causes of fainting episodes, such as vasovagal syncope or orthostatic hypotension, may initially be misattributed to accessory pathway-mediated arrhythmias, particularly when the clinical history includes episodic palpitations or syncope. However, these conditions lack the electrophysiological evidence of accessory pathways seen in FVP or WPW syndrome and are better characterized by autonomic or reflex-mediated pathophysiology^[13]^. The differentiation of FVP from other accessory pathways, arrhythmias, and conditions relies heavily on advanced diagnostic techniques, including intracardiac EPS, signal-averaged ECG, and imaging modalities such as cardiac MRI. EPS, in particular, allows for the direct measurement of conduction times and pathway localization, offering definitive diagnostic clarity. The presence of FVP is confirmed by its unique electrophysiological properties, such as retrograde conduction during ventricular pacing and the absence of preexcitation during sinus rhythm^[14]^.

## Management approaches

### Pharmacological management

This is a cornerstone in treating WPW syndrome, particularly for controlling arrhythmias and managing symptoms. Antiarrhythmic medications modify cardiac electrophysiology and suppress abnormal electrical activity to prevent or terminate arrhythmias. These medications exert their effects through various mechanisms, including sodium channel blockade, beta-adrenergic receptor antagonism, potassium channel modulation, and calcium channel blockade^[10]^. The choice of antiarrhythmic medication depends on the type of arrhythmia, underlying cardiac pathology, patient characteristics, and individual response to therapy. Sodium channel blockers, such as procainamide and flecainide, are commonly used to suppress AVRT and other supraventricular arrhythmias in individuals with WPW syndrome. These medications work by slowing conduction through the accessory pathway and reducing the likelihood of reentrant circuits, thereby terminating arrhythmias and restoring sinus rhythm^[11]^. Procainamide, in particular, is effective in prolonging the refractory period of accessory pathways, thereby preventing reentrant arrhythmias. Its efficacy is especially noted in acute settings where rapid arrhythmia control is necessary. However, using sodium channel blockers requires careful consideration of the patient’s cardiac function and the potential for proarrhythmic effects, especially in those with pre-existing heart conditions or structural abnormalities^[12]^. Beta-adrenergic receptor antagonists, such as metoprolol and propranolol, are frequently employed to control heart rate and alleviate symptoms in individuals with WPW syndrome, particularly during episodes of AF. Beta-blockers reduce myocardial excitability and suppress the effects of catecholamines, which can exacerbate arrhythmias. By slowing conduction through the accessory pathway, beta-blockers help prevent arrhythmia recurrence. They are particularly effective in patients experiencing palpitations, chest pain, or other symptoms of increased sympathetic activity. These agents are often well-tolerated but can cause bradycardia or hypotension, particularly in patients with concomitant cardiac conditions^[13]^. Potassium channel modulators, such as amiodarone and sotalol, are utilized in individuals with refractory arrhythmias or those at high risk of recurrent episodes despite treatment with other antiarrhythmic medications. Amiodarone, a complex antiarrhythmic agent, exhibits multiple electrophysiological effects, including sodium and potassium channel blockade, calcium channel inhibition, and beta-adrenergic receptor antagonism. This broad spectrum of activity makes it effective against various arrhythmias, including AF and ventricular arrhythmias. However, amiodarone is associated with significant side effects, such as thyroid dysfunction, pulmonary toxicity, and hepatic impairment, necessitating regular monitoring and cautious use^[14]^. Sotalol, which combines beta-adrenergic receptor blockade with potassium channel blockade, provides both rate and rhythm control in individuals with WPW syndrome. While it is generally well-tolerated, sotalol can prolong the QT interval, increasing the risk of torsades de pointes, a potentially life-threatening ventricular arrhythmia. Calcium channel blockers, including verapamil and diltiazem, are employed to manage heart rate and symptoms in individuals with WPW syndrome, especially during episodes of AF or atrial flutter. These agents work by slowing conduction through the accessory pathway and suppressing AV nodal conduction, thereby reducing the ventricular response rate and preventing hemodynamic compromise. Verapamil, in particular, is effective in individuals with rapid ventricular rates during AF or other supraventricular arrhythmias. However, the use of calcium channel blockers must be approached with caution in patients with heart failure or significant left ventricular dysfunction, as these medications can exacerbate heart failure symptoms^[15]^. Despite their efficacy in suppressing arrhythmias and controlling symptoms, antiarrhythmic medications present several limitations and potential risks in the management of WPW syndrome. One significant concern is the risk of proarrhythmia, a paradoxical effect where antiarrhythmic medications may exacerbate existing arrhythmias or precipitate new arrhythmias. This risk is particularly relevant in individuals with underlying structural heart disease or prolonged QT intervals. Proarrhythmic effects may result from drug-induced alterations in cardiac conduction, refractoriness, or automaticity, leading to severe ventricular arrhythmias such as torsades de pointes or VF^[16]^. Another limitation of pharmacological management in WPW syndrome is the variable response to antiarrhythmic medications among patients. The response to drug therapy can be influenced by factors such as the specific type of arrhythmia, the properties of the accessory pathway, patient characteristics, and concurrent medications or comorbidities. Some individuals may experience complete symptom resolution with a single medication, while others may require combination therapy or escalation to more potent antiarrhythmic agents to achieve adequate control. This variability necessitates a personalized approach to treatment, where the selection and titration of medications are tailored to the individual patient’s clinical profile and therapeutic response^[17]^. Additionally, antiarrhythmic medications may have adverse effects or drug interactions that limit their use in individuals with WPW syndrome. Common side effects of these medications include bradycardia, hypotension, fatigue, dizziness, nausea, and visual disturbances. These side effects can impact the patient’s quality of life and may necessitate dose adjustments or discontinuation of therapy. Drug interactions, particularly with other cardiovascular medications such as beta-blockers, calcium channel blockers, or anticoagulants, require careful monitoring and management to prevent adverse outcomes. For instance, the concomitant use of multiple agents that affect cardiac conduction can increase the risk of severe bradycardia or AV block^[18]^. Despite these challenges, pharmacological management remains a vital component of the treatment algorithm for WPW syndrome, especially in individuals with symptomatic arrhythmias or those at increased risk of adverse cardiac events. Antiarrhythmic medications provide significant relief from symptoms, reduce the frequency and severity of arrhythmia episodes, and enhance the quality of life for many patients. The careful selection of appropriate medications, individualization of therapy, and close monitoring for adverse effects are essential to optimize treatment outcomes and minimize risks in patients with WPW syndrome^[23,35,36]^.

### Invasive management approaches

During catheter ablation, specialized catheters are advanced into the heart through peripheral blood vessels, typically via the femoral vein. The catheters are guided under fluoroscopic and electroanatomic mapping guidance to the site of the accessory pathway, which is identified based on pre-procedural ECG findings, EPS, and intracardiac electrograms. Radiofrequency energy is then delivered through the catheter tip to the targeted tissue, creating localized thermal injury and ablating the accessory pathway^[37]^. Catheter ablation aims to achieve complete electrical isolation of the accessory pathway while preserving normal cardiac conduction. Catheter ablation techniques vary depending on the accessory pathway’s location, properties, and characteristics. In individuals with manifest WPW syndrome, where the accessory pathway conducts impulses anterogradely from atria to ventricles, ablation is typically performed at the site of the pathway’s insertion into the ventricles. This may involve targeting the AV annulus, the bundle of His region, or specific anatomical landmarks such as the coronary sinus ostium or the inferoseptal or posteroseptal regions of the left ventricle^[38,39]^. Ablation at these sites aims to interrupt the conduction pathway of the accessory pathway and prevent reentrant arrhythmias. In individuals with concealed WPW syndrome, where the accessory pathway conducts impulses retrogradely from ventricles to atria, ablation is typically performed at the site of the pathway’s origin in the ventricles. This may involve targeting specific anatomical structures such as the ventricular septum, the left or right ventricular free wall, or the epicardial surface of the heart^[40]^. Ablation at these sites aims to interrupt the retrograde conduction pathway of the accessory pathway and prevent reentrant arrhythmias. The success rates of catheter ablation in WPW syndrome are high, with most individuals achieving complete elimination of the accessory pathway and resolution of arrhythmias. Success rates vary depending on the location, properties, and characteristics of the accessory pathway, operator experience, and procedural technique. Reported success rates for catheter ablation in WPW syndrome range from 85% to 95%, with long-term freedom from arrhythmia recurrence^[41]^. Repeated ablation procedures may be required in some individuals due to accessory pathway reconnection or arrhythmia recurrence. Complications of catheter ablation in WPW syndrome are relatively rare but may include vascular injury, cardiac perforation, tamponade, AV block, coronary artery injury, stroke, and thromboembolism^[42]^. The risk of complications is influenced by factors such as patient age, comorbidities, procedural complexity, and operator experience. Procedural complications are typically managed conservatively with supportive measures such as fluid resuscitation, vasopressors, blood transfusion, or pericardiocentesis, as necessary. The overall risk of complications is low, and the benefits of catheter ablation in preventing arrhythmias and improving quality of life outweigh the potential risks in most individuals with WPW syndrome^[43]^. In addition to catheter ablation, surgical options may be considered in individuals with WPW syndrome, particularly those with complex or refractory arrhythmias, structural heart disease, or anatomical factors that preclude endocardial ablation. Surgical options for WPW syndrome include various approaches such as epicardial ablation, accessory pathway resection, maze procedure, and surgical cryoablation. Epicardial ablation involves accessing the heart through a surgical incision or thoracoscopic approach and ablating the accessory pathway from the heart’s outer surface^[44]^. This approach may be preferred in individuals with deep or inaccessible accessory pathways, those with prior failed endocardial ablation, or those with associated structural heart disease requiring concomitant surgical intervention^[5]^. Epicardial ablation provides direct visualization of the myocardium and allows for precise targeting of the accessory pathway, with high success rates and low risk of complications. Accessory pathway resection involves surgically excising the abnormal myocardial tissue responsible for the accessory pathway, typically during open-heart surgery, such as mitral valve repair, coronary artery bypass grafting, or congenital heart defect repair^[45]^. This approach may be indicated in individuals with complex or multiple accessory pathways, those with associated structural heart disease requiring surgical intervention, or those with contraindications to catheter ablation^[6]^. Accessory pathway resection eliminates the risk of arrhythmia recurrence and may provide a long-term cure in selected individuals with WPW syndrome. The maze procedure is a surgical technique originally developed for treating AF. Still, it may also be used in individuals with WPW syndrome, particularly those with coexisting arrhythmias or structural heart disease. The maze procedure involves creating a series of surgical incisions or ablations on the atria to disrupt abnormal electrical pathways and create a maze-like pattern of scar tissue that redirects electrical impulses and restores normal sinus rhythm^[46]^. This approach may be indicated in individuals with refractory arrhythmias, those with multiple or complex accessory pathways, or those with associated structural heart disease requiring surgical intervention^[7]^. The maze procedure effectively restores sinus rhythm and reduces arrhythmia recurrence in individuals with WPW syndrome. Surgical cryoablation is another option for the treatment of WPW syndrome, particularly in individuals with deep or inaccessible accessory pathways or those with contraindications to radiofrequency ablation^[15]^. Cryoablation involves delivering freezing temperatures to the targeted tissue through a cryoprobe, resulting in cellular injury and necrosis. This approach may be performed either epicardially or endocardially, depending on the location and characteristics of the accessory pathway. Cryoablation provides an alternative to radiofrequency ablation and may be associated with lower rates of complications such as vascular injury or cardiac perforation^[8]^. However, long-term outcomes and efficacy compared to radiofrequency ablation require further investigation.

### Clinical practice guidelines

The American College of Cardiology/American Heart Association (ACC/AHA) Guidelines provide comprehensive recommendations for diagnosing, managing, and treating arrhythmias, including WPW syndrome. These guidelines offer evidence-based guidance on risk assessment, pharmacological therapy, catheter ablation techniques, and long-term follow-up care. They emphasize the importance of individualized treatment strategies tailored to the specific needs and characteristics of each patient with WPW syndrome^[1]^. The ACC/AHA guidelines highlight the role of risk stratification in identifying individuals with WPW syndrome who are at increased risk of adverse cardiac events such as SVT, AF, or sudden cardiac arrest. Risk factors for arrhythmia recurrence and complications include the presence of symptomatic WPW syndrome, history of syncope or palpitations, accessory pathway properties (e.g. location, conduction properties), and coexisting structural heart disease. Clinicians are advised to assess these risk factors systematically and consider them when developing management strategies for individuals with WPW syndrome^[1]^. Regarding pharmacological therapy, the ACC/AHA guidelines recommend antiarrhythmic medications such as beta-blockers, calcium channel blockers, or procainamide for symptom control and arrhythmia prevention in individuals with WPW syndrome. These medications aim to reduce the frequency and severity of arrhythmia episodes, control heart rate during SVT, and minimize the risk of arrhythmia-related complications. However, when selecting antiarrhythmic agents, factors such as drug efficacy, safety, tolerability, and potential adverse effects on cardiac function should be considered^[1]^. Catheter ablation is considered the first-line treatment for individuals with symptomatic WPW syndrome or those at increased risk of arrhythmia recurrence. The ACC/AHA guidelines recommend catheter ablation as a curative approach to eliminate the accessory pathway and prevent arrhythmia recurrence. Ablation procedures are generally safe and effective, with high success rates and low rates of procedural complications when performed by experienced electrophysiologists in specialized centers. Repeated ablation procedures may be necessary for some individuals due to accessory pathway recurrence or arrhythmia persistence^[1]^.The European Society of Cardiology (ESC) Guidelines on managing arrhythmias offer evidence-based recommendations for treating WPW syndrome, focusing on European populations. These guidelines provide guidance on risk assessment, antiarrhythmic therapy, catheter ablation techniques, and management of arrhythmias during pregnancy. They emphasize the importance of multidisciplinary care and individualized treatment strategies tailored to the specific needs and preferences of each patient with WPW syndrome^[2]^. The ESC guidelines highlight the role of imaging modalities such as cardiac MRI and CT in diagnosing and characterizing accessory pathways in individuals with WPW syndrome. These imaging techniques offer detailed anatomical information, identify accessory pathways, and assess myocardial tissue characteristics without requiring invasive catheterization procedures. Advanced imaging techniques such as late gadolinium enhancement MRI and three-dimensional (3D) CT reconstruction facilitate pre-procedural planning for catheter ablation and improve procedural outcomes^[2]^. Regarding pharmacological therapy, the ESC guidelines recommend beta-blockers, calcium channel blockers, or antiarrhythmic agents such as flecainide or propafenone for symptom control and arrhythmia prevention in individuals with WPW syndrome. These medications aim to suppress arrhythmias, control heart rate, and reduce the frequency and severity of episodes, thereby improving symptoms and quality of life. The selection of antiarrhythmic agents should consider factors such as drug efficacy, safety, tolerability, and potential interactions with concomitant medications^[2]^. Catheter ablation is recommended as the preferred treatment modality for individuals with symptomatic WPW syndrome or those at increased risk of arrhythmia recurrence. The ESC guidelines emphasize the importance of catheter ablation as a curative approach to eliminate the accessory pathway and prevent arrhythmia recurrence. Ablation procedures are safe and effective when performed by experienced electrophysiologists in specialized centers, with high success rates and low rates of procedural complications. Long-term follow-up and monitoring are essential to assess treatment efficacy, detect arrhythmia recurrence, and evaluate for potential complications^[2]^. Pediatric guidelines offer specific recommendations for the management of arrhythmias in children and adolescents, including those with WPW syndrome. These guidelines address risk assessment, treatment options, and long-term monitoring tailored to pediatric patients’ unique physiological and developmental considerations. They emphasize the importance of early detection, prompt intervention, and multidisciplinary care in optimizing outcomes for children with WPW syndrome^[3]^. The pediatric guidelines highlight the role of genetic testing in identifying underlying genetic variants associated with WPW syndrome and other congenital cardiac conditions. Genetic testing can provide valuable insights into disease pathogenesis, inheritance patterns, and family screening strategies. Additionally, the guidelines emphasize the importance of close monitoring and long-term follow-up in pediatric patients with WPW syndrome to assess cardiac function, detect arrhythmia recurrence, and evaluate potential complications^[3]^. Electrophysiology society guidelines published by professional societies such as the Heart Rhythm Society or the European Heart Rhythm Association provide expert consensus recommendations on diagnosing and managing arrhythmias, including WPW syndrome. Based on the latest evidence and expert consensus, these guidelines offer practical insights into procedural techniques, ablation strategies, and peri-procedural care. They guide patient selection, procedural considerations, and post-procedural management to optimize outcomes and minimize complications^[4]^. Table 3 shows various aspects of management for WPW syndrome.

**Table 3:** Various aspects of management for Wolff–Parkinson–White (WPW) syndrome

Aspect of management	American College of Cardiology/American Heart Association (ACC/AHA) Guidelines	European Society of Cardiology (ESC) Guidelines	Pediatric guidelines	Electrophysiology society guidelines
Risk assessment	- Evaluate for symptoms (e.g. palpitations, syncope)	- Assess symptoms and risk factors for arrhythmia recurrence	- Consider family history of arrhythmias and congenital heart disease	- Assess symptoms, accessory pathway properties, coexisting heart disease, and risk factors for arrhythmia recurrence
Diagnostic evaluation	- Perform electrocardiography (ECG) to assess for preexcitation patterns	- Use ECG, Holter monitoring, exercise stress testing, echocardiography, and electrophysiological studies	- Utilize ECG, Holter monitoring, exercise stress testing, echocardiography, and electrophysiological studies	- Employ ECG, Holter monitoring, exercise stress testing, echocardiography, and electrophysiological studies
Pharmacological therapy	- Consider beta-blockers, calcium channel blockers, or procainamide for symptom control	- Recommend beta-blockers, calcium channel blockers, or flecainide/propafenone for symptom control	- Use beta-blockers or calcium channel blockers for symptom control	- Recommend beta-blockers or calcium channel blockers for symptom control
Catheter ablation	- Offer catheter ablation as first-line treatment for symptomatic or high-risk individuals	- Recommend catheter ablation as the preferred treatment modality for symptomatic individuals	- Consider catheter ablation for symptomatic individuals with high-risk accessory pathways	- Offer catheter ablation as first-line treatment for symptomatic or high-risk individuals
Imaging modalities	- Utilize cardiac MRI, CT, or echocardiography for anatomical assessment	- Employ cardiac MRI, CT, or echocardiography for anatomical assessment	- Use echocardiography for anatomical assessment	- Use echocardiography for anatomical assessment
Antiarrhythmic medications	- Consider flecainide or propafenone for acute termination of arrhythmias	- Recommend flecainide or propafenone for acute termination of arrhythmias	- Consider flecainide or propafenone for acute termination of arrhythmias	- Recommend flecainide or propafenone for acute termination of arrhythmias
Pregnancy management	- Assess the risk of arrhythmia exacerbation and adverse maternal/fetal outcomes	- Monitor for arrhythmia recurrence, hemodynamic compromise, and fetal well-being during pregnancy	- Evaluate arrhythmia risk and impact on pregnancy outcomes	- Monitor for arrhythmia recurrence, hemodynamic compromise, and fetal well-being during pregnancy
Complications and prognosis	- Educate patients about potential complications such as arrhythmias, heart failure, and sudden cardiac arrest	- Counsel patients about the risk of arrhythmia recurrence, heart failure, and sudden cardiac death	- Inform families about potential complications and long-term prognosis	- Educate patients about potential complications such as arrhythmias, heart failure, and sudden cardiac arrest

This table compares management recommendations from different guidelines, including risk assessment, diagnostic evaluation, pharmacological therapy, catheter ablation, imaging modalities, antiarrhythmic medications, pregnancy management, and complications/prognosis. [Authors Creations.]

## Lifestyle modifications and patient education

Patient education is vital in managing WPW syndrome, focusing on understanding the condition’s nature, underlying mechanisms, and potential complications. Educating patients about the abnormal accessory pathway’s role in predisposing them to arrhythmias, such as SVT and AF, empowers them to recognize symptoms early and seek timely medical attention. Patients should be advised to minimize or avoid potential triggers for arrhythmias, including excessive alcohol, caffeine, tobacco, and illicit drugs. Encouraging a heart-healthy lifestyle is essential. Patients should engage in regular aerobic exercise (walking, cycling, swimming) as tolerated while being mindful of exercise intensity and duration to prevent arrhythmia onset. Guidelines should be personalized based on individual clinical characteristics and arrhythmia risk profiles. A balanced diet rich in fruits, vegetables, whole grains, lean proteins, and healthy fats while limiting sodium, saturated fats, and sugars can help manage blood pressure and weight, reducing arrhythmia risk. Maintaining a healthy weight and effectively managing comorbid conditions (hypertension, diabetes, dyslipidemia) is crucial. Patients should adhere to prescribed medications to prevent arrhythmia recurrence. Addressing psychosocial factors is important; healthcare providers should offer emotional support and resources to help patients manage anxiety and stress related to their condition. Educating patients on recognizing signs of arrhythmias (palpitations, dizziness, chest pain, shortness of breath, or syncope) and the importance of regular follow-up appointments is essential. Patients should be encouraged to seek medical attention if they experience symptoms that are concerning.

## Special populations

In pediatric patients, WPW syndrome presents unique challenges and considerations compared to adults due to differences in cardiac anatomy, arrhythmia mechanisms, and management strategies^[47]^. WPW syndrome is characterized by an abnormal accessory pathway that creates a preexcitation circuit, allowing electrical impulses to bypass the normal AV node conduction pathway^[48]^. In pediatric patients, the presentation of WPW syndrome can vary widely, from asymptomatic cases detected incidentally on an ECG to symptomatic patients experiencing frequent or severe arrhythmias. The clinical management of WPW syndrome in children requires careful consideration of the patient’s age, comorbidities, and potential for future arrhythmias^[34]^. Pediatric patients with WPW syndrome can present with a range of symptoms, including palpitations, syncope, or chest pain.

In some cases, the syndrome may be discovered during evaluation for other conditions or as part of a routine screening for familial arrhythmias^[49]^. The approach to managing WPW syndrome in pediatric patients often includes clinical assessment, risk stratification, and therapeutic interventions tailored to the individual patient’s needs. One significant aspect of WPW syndrome in pediatric patients is the association with congenital cardiac anomalies, such as Ebstein anomaly and double accessory pathways^[47]^. Ebstein anomaly is a rare congenital heart defect associated with WPW syndrome, characterized by malformation of the tricuspid valve, which can lead to right atrial enlargement and impaired right ventricular function. This altered cardiac anatomy complicates arrhythmia management in patients with WPW, as accessory pathways may have atypical locations and conduction properties, making catheter ablation more challenging. In cases of WPW syndrome, a multidisciplinary approach involving pediatric cardiologists, electrophysiologists, and cardiac surgeons is often necessary to optimize treatment outcomes.

Additionally, double accessory pathways can occur in pediatric patients with WPW, leading to complex arrhythmias such as AVRT and AF. Effective management requires detailed electrophysiological evaluation to map the pathways for ablation accurately. Catheter ablation is a key treatment for WPW syndrome, particularly when medications are ineffective. The procedure must be adapted for pediatric patients due to their smaller cardiac structures and unique arrhythmia mechanisms, focusing on minimizing risks and ensuring successful outcomes^[50]^.

Management of WPW syndrome during pregnancy focuses on minimizing arrhythmia recurrence, optimizing maternal cardiac function, and ensuring fetal safety. Antiarrhythmic medications, such as beta-blockers or calcium channel blockers, may be considered for symptomatic patients or those at high risk for arrhythmias. However, certain medications, like amiodarone, are contraindicated due to potential teratogenic effects. Catheter ablation is the preferred treatment for symptomatic or high-risk WPW syndrome during pregnancy^[51]^. This curative procedure can eliminate the accessory pathway and prevent arrhythmia recurrence. While generally safe and effective, the timing of the ablation should be carefully considered. It is typically avoided in the first trimester due to teratogenic risks. Still, if symptoms are severe, it may be performed in the second or third trimester under close maternal-fetal monitoring. In addition to medical management, lifestyle modifications are essential. Pregnant women with WPW syndrome should avoid caffeine, alcohol, tobacco, and illicit drugs, which can exacerbate arrhythmias. Adequate hydration, stress management, and rest are crucial for maintaining cardiac function and reducing arrhythmia risk^[52]^. Regular maternal-fetal monitoring, including obstetric evaluations and cardiac assessments, is necessary to ensure maternal and fetal well-being. Collaboration among obstetricians, cardiologists, and maternal-fetal medicine specialists is vital to optimize outcomes. In cases of arrhythmia exacerbation or hemodynamic instability, timely intervention may involve pharmacological therapy, catheter ablation, or emergent cardioversion to safeguard both mother and fetus.

In patients with WPW and structural heart disease, arrhythmia presentations may be complex due to overlapping electrical and structural abnormalities. For instance, arrhythmias in those with WPW and atrial septal defect (ASD) may arise from atrial stretch and remodeling, while in patients with WPW and HCM, triggers may include myocardial hypertrophy and fibrosis. Diagnosis requires a comprehensive evaluation of symptoms, medical history, ECG findings, and imaging studies such as echocardiography or cardiac MRI to assess cardiac anatomy and function. Structural heart disease can complicate ECG interpretation, with potential coexisting conditions like bundle branch blocks or ventricular hypertrophy masking preexcitation patterns. Management strategies focus on minimizing arrhythmia risk and optimizing cardiac function. Pharmacological options, including beta-blockers, calcium channel blockers, and procainamide, may be used judiciously, considering the implications of the underlying structural conditions^[51–53]^. Catheter ablation is preferred for treating WPW, effectively eliminating accessory pathways; however, it can be technically challenging due to anatomical variations and hemodynamic considerations. Risk assessment for procedural complications such as AV block or coronary artery injury is crucial, especially when accessory pathways are near vital structures. In addition to pharmacotherapy and ablation, lifestyle modifications are essential. Patients should be advised to avoid caffeine, alcohol, tobacco, and illicit drugs, all of which can exacerbate arrhythmias. Emphasizing heart-healthy behaviors – such as regular exercise, balanced nutrition, and stress management – can optimize cardiac health. Regular follow-ups with cardiologists or electrophysiologists are necessary for monitoring and adjusting treatment. Patient education is also vital, empowering individuals to understand their conditions and adhere to treatment plans effectively^[53]^.

## Complications and prognosis

WPW syndrome is associated with several complications, primarily life-threatening arrhythmias, including AF, VF, and sudden cardiac arrest. SVT can degenerate into AF with rapid ventricular response, potentially leading to hemodynamic instability, syncope, or cardiac arrest. In patients with ventricular preexcitation, rapid conduction through accessory pathways can facilitate reentrant circuits, increasing the risk of VF and SCD. Prompt recognition and management of these arrhythmias are crucial to prevent adverse outcomes^[10–12]^. AF in WPW syndrome can occur spontaneously or be triggered by factors such as alcohol, caffeine, or emotional stress. This condition presents a significant risk for rapid ventricular response and hemodynamic compromise, particularly in pathways with high conduction velocities. Anticoagulation may be necessary in cases of WPW with AF to reduce thromboembolic risks, including stroke^[13–15]^. Structural heart abnormalities, such as ASDs, ventricular septal defects, Ebstein’s anomaly, or HCM, can also be associated with WPW syndrome, contributing to arrhythmia susceptibility and adverse cardiac events. The presence of underlying heart disease complicates management and increases the risk of arrhythmia recurrence, heart failure, or SCD. Close monitoring and evaluation of cardiac structure and function are essential to identify and address potential abnormalities^[16–18]^. Iatrogenic complications can arise from diagnostic or therapeutic interventions. While catheter ablation is a curative option for WPW syndrome, it carries risks, including AV block, coronary artery injury, or cardiac perforation. The incidence of these complications varies based on operator experience, procedural complexity, and patient characteristics. Careful pre-procedural planning and post-procedural follow-up are vital for minimizing risks and optimizing outcomes^[19,20,28]^.

Additionally, WPW syndrome can affect pregnancy outcomes, as arrhythmias and hemodynamic instability pose risks to both maternal and fetal health. Pregnant women with WPW require close monitoring during pregnancy, labor, and delivery to manage arrhythmias and potential complications effectively. Management strategies may involve pharmacological therapy, cardioversion, or catheter ablation tailored to the individual’s needs^[1,32,33]^. Despite these complications, the overall prognosis for WPW syndrome is generally favorable with appropriate treatment and risk stratification. Individuals with asymptomatic WPW and low-risk accessory pathways typically have an excellent prognosis, with a low likelihood of arrhythmia recurrence. However, those with symptomatic WPW, high-risk pathways, or coexisting structural heart disease may face a less favorable prognosis, necessitating closer monitoring and aggressive management to prevent complications^[21,22,54]^. Prognostic factors include age of onset, symptom severity, accessory pathway characteristics, and response to treatment. Long-term follow-up shows that individuals with WPW syndrome can lead normal lives with effective medical management, lifestyle modifications, and, in some cases, catheter ablation. Regular cardiac evaluations and adherence to treatment recommendations are essential for optimizing outcomes^[29–31]^.

## Recent advances and future research directions

Refinements in diagnostic tools, such as high-resolution ECG and advanced imaging techniques like cardiac MRI and CT, have enhanced the accuracy of accessory pathway localization. These technologies facilitate targeted therapeutic interventions. Sophisticated EPS now enable detailed mapping of the heart’s electrical activity, which is critical for identifying accessory pathway characteristics and planning appropriate therapeutic approaches^[20]^. Catheter ablation has become the first-line treatment for symptomatic WPW syndrome, with significant advancements improving success rates and reducing complications. High-power, short-duration radiofrequency (RF) ablation techniques minimize collateral damage and decrease procedure time compared to traditional methods.

Furthermore, 3D mapping systems provide real-time visualization of cardiac anatomy, enhancing catheter placement precision^[28]^. Cryoablation has also gained traction as an alternative, especially for pediatric patients and those with accessory pathways near vital cardiac structures. Its reversibility allows cryo-mapping to assess the efficacy of the ablation site before full cryotherapy, thereby reducing the risk of damage to critical structures like the AV node. Recent studies have shown comparable success rates between cryoablation and RF ablation, making it a viable option for specific patient populations^[32]^. The development of noninvasive risk stratification tools has further advanced WPW management. Techniques such as exercise testing and Holter monitoring are increasingly utilized to assess the risk of arrhythmia recurrence and SCD in asymptomatic patients. These methods help identify individuals who may benefit from aggressive interventions like prophylactic ablation.

Additionally, integrating genetic testing into risk stratification may allow personalized treatment strategies based on identified mutations associated with WPW syndrome^[33]^. Pharmacological management remains crucial for patients not eligible for catheter ablation or those experiencing arrhythmia recurrence. Recent advancements include optimizing antiarrhythmic agents such as flecainide and propafenone, which effectively suppress arrhythmias by blocking sodium channels and slowing conduction through the accessory pathway. Beta-blockers and calcium channel blockers are valuable in controlling ventricular rates during AF. However, careful monitoring is essential due to potential proarrhythmic effects^[1]^. Recognition of lifestyle modifications’ role in managing WPW syndrome has grown. Patients are advised to avoid triggers, such as excessive caffeine and alcohol, and to maintain a healthy lifestyle. Education on recognizing arrhythmia symptoms and understanding when to seek medical attention is critical, particularly for those at risk of SCD. The advent of wearable technology, including smartwatches and portable ECG devices, allows for real-time monitoring of cardiac rhythms, enabling timely interventions when arrhythmias are detected^[21]^. Future research aims to enhance the safety and efficacy of therapeutic interventions and deepen our understanding of WPW pathophysiology. Ongoing studies explore robotic-assisted catheter ablation and magnetic navigation systems to improve precision and consistency, potentially reducing operator dependence^[22]^. Gene therapy and regenerative medicine are also being investigated as treatments for WPW syndrome. Research is underway to identify the genetic basis of the condition and develop gene-editing technologies like CRISPR-Cas9 to correct underlying defects. Although still in the experimental phase, these approaches may offer a definitive cure by targeting the root causes of WPW syndrome. Stem cell therapy is also being explored to regenerate damaged cardiac tissues and restore normal conduction pathways^[54]^. Finally, integrating artificial intelligence (AI) and machine learning in WPW management represents an exciting frontier. AI algorithms can analyze extensive ECG recordings and clinical parameters datasets to identify patterns indicative of WPW syndrome. These technologies promise to improve diagnostic accuracy, predict arrhythmia recurrence risks, and guide personalized treatment plans. Furthermore, AI may assist in optimizing ablation strategies by predicting effective ablation sites and energy settings based on individual patient characteristics^[29]^.

## Long-term follow-up and monitoring

Long-term follow-up for patients with WPW syndrome is crucial for effective management, primarily to monitor arrhythmia recurrence and evaluate the efficacy of interventions. Regular clinical evaluations help assess the success of catheter ablation procedures, which are the primary treatment for symptomatic WPW syndrome^[2,3,5]^. The first follow-up visit is typically scheduled within 1–3 months post-ablation, with subsequent visits every 6–12 months. These visits include a thorough clinical assessment, symptom review, physical examination, and ECG testing to detect residual arrhythmias or conduction abnormalities. EPS are particularly important for high-risk patients, such as those with recurrent symptoms, multiple accessory pathways, or arrhythmias unresponsive to pharmacological therapy. EPS allows for detailed mapping of the heart’s electrical activity, helping clinicians evaluate the accessory pathway’s functionality and the effectiveness of prior treatments^[6–8]^. In cases where patients present with AF or other arrhythmias post-ablation, EPS can identify residual or new accessory pathways and guide subsequent treatment strategies, including repeat ablation or alternative therapies.

Beyond monitoring arrhythmia recurrence, long-term follow-up includes evaluating the overall cardiac health of WPW syndrome patients. These individuals are at risk for developing additional cardiac conditions, such as heart failure or structural heart disease, which arrhythmias or antiarrhythmic medications can exacerbate^[9–11]^. Regular echocardiographic evaluations and imaging studies may be necessary to assess cardiac function and detect structural changes over time. The integration of wearable technology and remote monitoring is becoming increasingly significant in managing WPW syndrome^[12–14]^. Devices like smartwatches and portable ECG monitors enable continuous heart rhythm monitoring, providing real-time data on arrhythmias and alerting patients and healthcare providers to potential issues. This proactive approach enhances patient engagement and facilitates timely interventions, reducing the risk of serious complications. The approach to follow-up can differ for asymptomatic or infrequently symptomatic patients, who may not need frequent visits or invasive testing if they are stable and show no significant arrhythmias on routine ECGs^[15–17]^. However, periodic evaluations should still be conducted to monitor for any underlying issues that could impact long-term health, typically through annual or biennial assessments. For high-risk patients, EPS is especially crucial for long-term monitoring. Those with a history of failed ablation or persistent arrhythmias may benefit from repeat EPS to evaluate the current status of accessory pathways and adjust treatment plans accordingly. Patient education is also a vital component of long-term follow-up. Patients should be informed about recognizing arrhythmia symptoms and when to seek medical attention. Lifestyle modifications, such as avoiding known arrhythmia triggers and maintaining a healthy lifestyle, can significantly improve long-term outcomes. Educating patients on the risks associated with WPW syndrome and the importance of adhering to treatment plans can enhance their quality of life and overall health management^[18–20]^.

## Concluding remarks

WPW syndrome presents a significant risk for arrhythmias, requiring careful diagnosis and management. Catheter ablation remains an effective treatment for symptomatic patients, while advancements in mapping and genetic studies continue to improve clinical outcomes. Further research is needed to enhance understanding of WPW’s genetic factors and to refine risk stratification and treatment approaches, ensuring better long-term care for affected individuals.

## Call to action

Healthcare professionals should prioritize early diagnosis and risk stratification in WPW syndrome to prevent life-threatening complications. To ensure optimal outcomes, leverage noninvasive diagnostic tools and consider catheter ablation for high-risk patients.

1. WPW syndrome can cause dangerous heart rhythms and sudden cardiac events.
2. Risk assessment helps identify patients needing close monitoring or treatment.
3. Management includes medications, ablation, or lifestyle adjustments for safety.

## Data Availability

This published article and its supplementary information files include all data generated or analyzed during this study.

## References

[1] Chhabra L Goyal A Benham MD. Wolff-Parkinson-White syndrome. [Updated 2023 Aug 7]. In: StatPearls [Internet]. Treasure Island (FL): StatPearls Publishing; 2024. https://www.ncbi.nlm.nih.gov/books/NBK554437/. 32119324

[2] Yildirim I Ozer S Karagoz T. Clinical and electrophysiological evaluation of pediatric Wolff-Parkinson-White patients. Anatol J Cardiol 2015;15:485–90. 26006136 10.5152/akd.2014.5462 PMC5779142

[3] Chang YT Chung CM. A novel and simple algorithm using surface electrocardiogram that localizes accessory conduction pathway in Wolff-Parkinson-White syndrome in pediatric patients. Acta Cardiol Sin 2019;35:493–500. 31571798 10.6515/ACS.201909_35(5).20190312A PMC6760133

[4] Baek SM Song MK Uhm JS. New algorithm for accessory pathway localization focused on screening septal pathways in pediatric patients with Wolff-Parkinson-White syndrome. Heart Rhythm 2020;17:2172–79. 32681992 10.1016/j.hrthm.2020.07.016

[5] Taguchi N Yoshida N Inden Y. A simple algorithm for localizing accessory pathways in patients with Wolff-Parkinson-White syndrome using only the R/S ratio. J Arrhythmia 2014;30:439–43.

[6] Meyer L Stubbs B Fahrenbruch C. Incidence, causes, and survival trends from cardiovascular-related sudden cardiac arrest in children and Young adults 0 to 35 years of age: a 30-year review. Circulation 2012;126:1363–72. 22887927 10.1161/CIRCULATIONAHA.111.076810

[7] Davenport ED Rupp KAN Palileo E. Asymptomatic Wolff-Parkinson-White pattern ECG in USAF aviators. Aerosp Med Hum Perform 2017;88:56–60. 28061924 10.3357/AMHP.4569.2017

[8] Collins KK Pass RH Aziz PF. Loss of ventricular preexcitation during noninvasive testing does not exclude high-risk accessory pathways: a multicenter study of WPW in children. Heart Rhythm 2020;17:1729–37. 32497761 10.1016/j.hrthm.2020.05.035

[9] Kiger ME Mccanta AC Tong S. Intermittent versus persistent Wolff-Parkinson-White syndrome in children: electrophysiologic properties and clinical outcomes. Pacing Clin Electrophysiol 2016;39:14–20. 26256551 10.1111/pace.12732

[10] Robles AG Palamà Z Pernat A. Intermittent ventricular pre-excitation in symptomatic adults: always a marker of low risk. Pacing Clin Electrophysiol 2023;46:1049–55. 37527153 10.1111/pace.14798

[11] Pappone C Vicedomini G Manguso F. Wolff-Parkinson-White syndrome in the era of catheter ablation insights from a registry study of 2169 patients. Circulation 2014;130:811–19. 25052405 10.1161/CIRCULATIONAHA.114.011154

[12] Raposo D António N Andrade H. Management of asymptomatic Wolff–Parkinson–White pattern in young patients: has anything changed. Pediatr Cardiol 2019;40:892–900. 31069431 10.1007/s00246-019-02110-6

[13] Delise P Sciarra L. Sudden cardiac death in patients with ventricular preexcitation. Card Electrophysiol Clin 2020;12:519–25. 33162000 10.1016/j.ccep.2020.08.002

[14] Apfel G Choi NH Silver ES. Assessing the utility of atrial fibrillation induction to risk stratify children with Wolff-Parkinson-White syndrome. Cardiol Young 2023;13:1–5. 10.1017/S1047951123001415 37309199

[15] Etheridge SP Gakenheimer-Smith L Asaki SY. Asymptomatic Wolff-Parkinson-White syndrome: an ounce of prevention is worth the risk of Cure. Curr Cardiol Rep 2023;25:543–51. 37115433 10.1007/s11886-023-01879-6

[16] Pelliccia A Sharma S Gati S. 2020 ESC Guidelines on sports cardiology and exercise in patients with cardiovascular disease. Eur Heart J 2021;42:17–96. 32860412 10.1093/eurheartj/ehaa605

[17] Vijay Y Sanjeev T Mani GR. A Wolff-Parkinson-White (WPW) electrocardiographic pattern in asymptomatic patient—state-of-the-art-review. J Cardiol Cardiovasc Med 2022;7:45–52.

[18] Kugler JD Danford DA Houston KA. Pediatric radiofrequency catheter ablation registry success, fluoroscopy time, and complication rate for supraventricular tachycardia: comparison of early and recent eras. J Cardiovasc Electrophysiol 2002;13:336–41. 12033349 10.1046/j.1540-8167.2002.00336.x

[19] Brado J Hochadel M Senges J. Outcomes of ablation in Wolff-Parkinson-White-syndrome: data from the German ablation registry. Int J Cardiol 2021;323:106–12. 32890614 10.1016/j.ijcard.2020.08.102

[20] Sherdia AFIA Abdelaal SA Hasan MT. The success rate of radiofrequency catheter ablation in Wolff-Parkinson-White-syndrome patients: a systematic review and meta-analysis. Indian Heart J 2023;75:98–107. 36758831 10.1016/j.ihj.2023.02.001 PMC10123428

[21] Vătășescu RG Paja CS Șuș I. Wolf-Parkinson-White syndrome: diagnosis, risk assessment, and therapy-an update. Diagnostics (Basel) 2024;14:296. 38337810 10.3390/diagnostics14030296 PMC10855590

[22] Elendu C Ogwu NP Okatta AU. Global research progress on radiofrequency ablation in cardiology. Ann Med Surg 2025;87:725–47. 10.1097/MS9.0000000000002858 PMC11918750 40110263

[23] Rao AL Salerno JC Asif IM. Evaluation and management of Wolff-Parkinson-White in athletes. Sports Health 2014;6:326–32. 24982705 10.1177/1941738113509059 PMC4065555

[24] Godugu S Sinha T Pradeepan M. Unraveling the enigma of aortic dissection: from genetics to innovative therapies. Cureus 2024;16:e57803. 38721226 10.7759/cureus.57803 PMC11077317

[25] Svendsen JH Dagres N Dobreanu D. Scientific initiatives committee, European Heart Rhythm Association. Current strategy for treatment of patients with Wolff-Parkinson-White syndrome and asymptomatic preexcitation in Europe: European Heart Rhythm Association Survey. Europace 2013;15:750–53. 23625944 10.1093/europace/eut094

[26] Al-Khatib SM Arshad A Balk EM. Risk stratification for arrhythmic events in patients with asymptomatic pre-excitation: a systematic review for the 2015 ACC/AHA/HRS guideline for the management of adult patients with supraventricular tachycardia: a report of the American College of Cardiology/American Heart Association Task Force on clinical practice guidelines and the heart rhythm society. J Am Coll Cardiol 2016;67:1624–38. 26409260 10.1016/j.jacc.2015.09.018

[27] Hlyan NP Arif T Jaufar SS. From sugar spikes to pressure peaks: navigating the world of diabetes, hypertension, obesity, and kidney health. Cureus 2024;16:e57241. 38686257 10.7759/cureus.57241 PMC11056813

[28] Krause U Paul T Della Bella P. Pediatric catheter ablation at the beginning of the 21st century: results from the European Multicenter Pediatric Catheter Ablation Registry ‘EUROPA.’ Eurospace 2021;23:431–40. 10.1093/europace/euaa325 33227133

[29] Munger TM Packer DL Hammill SC. A population study of the natural history of Wolff-Parkinson-White syndrome in Olmsted county, Minnesota, 1953-1989. Circulation 1993;87:866–73. 8443907 10.1161/01.cir.87.3.866

[30] Timmermans C Smeets JL Rodriguez LM. Aborted sudden death in the Wolff-Parkinson-White syndrome. Am J Cardiol 1995;76:492–94. 7653450 10.1016/s0002-9149(99)80136-2

[31] Wolff L Parkinson J White PD. Bundle-branch block with short P-R interval in healthy young people prone to paroxysmal tachycardia. 1930. Ann Noninvasive Electrocardiol 2006;11:340–53. 17040283 10.1111/j.1542-474X.2006.00127.x PMC6932258

[32] Borregaard R Lukac P Gerdes C. Radiofrequency ablation of accessory pathways in patients with the Wolff-Parkinson-White syndrome: the long-term mortality and risk of atrial fibrillation. Europace 2015;17:117–22. 25013013 10.1093/europace/euu176

[33] Bunch TJ May HT Bair TL. Long-term natural history of adult Wolff–Parkinson–White syndrome patients treated with and without catheter ablation. Circ Arrhythmia Electrophysiol 2015;8:1465–71. 10.1161/CIRCEP.115.003013 26480930

[34] Brugada J Katritsis DG Arbelo E. 2019 ESC Guidelines for the management of patients with supraventricular tachycardia. The Task Force for the management of patients with supraventricular tachycardia of the European Society of Cardiology (ESC). Eur Heart J 2019;41:655–720. 10.1093/eurheartj/ehz467 31504425

[35] Kumar S Khatri M Kumar S. Comparative efficacy and safety profiles of high-power, short-duration and low-power, long-duration radiofrequency ablation in atrial fibrillation: a systematic review and meta-analysis. J Innov Card Rhythm Manag 2023;14:5514–27. 37492695 10.19102/icrm.2023.14072 PMC10364668

[36] Smith RF. The Wolff-Parkinson-White syndrome as an aviation risk. Circulation 1964;29:672–79. 14153940

[37] Chung K-Y Walsh TJ Massie E. Wolff-Parkinson-White syndrome. Am Heart J 1965;69:116–33. 14253091 10.1016/0002-8703(65)90224-3

[38] Islam R Ahmed M Ullah W. Effect of caffeine in hypertension. Curr Probl Cardiol 2023;48:101892. 37394201 10.1016/j.cpcardiol.2023.101892

[39] Krahn AD Manfreda J Tate RB. The natural history of electrocardiographic preexcitation in men: the Manitoba follow-up study. Ann Intern Med 1992;116:456–60. 1739235 10.7326/0003-4819-116-6-456

[40] Page RL Joglar JA Caldwell MA. 2015 ACC/AHA/HRS guideline for the management of adult patients with supraventricular tachycardia: executive summary: a report of the American College of Cardiology/American Heart Association Task force on clinical practice guidelines and the heart rhythm society. Circulation 2016;133:e471–e505. 26399662 10.1161/CIR.0000000000000310

[41] Khatri M Kumar S Mahfooz K. Clinical outcomes of polymer-free versus polymer-coated drug-eluting stents in patients with coronary artery disease: a systematic review and meta-analysis. Cureus 2023;15:e38215. 37252538 10.7759/cureus.38215 PMC10224769

[42] Vidaillet HJ Pressley JC Henke E. Familial occurrence of accessory atrioventricular pathways (preexcitation syndrome). N Engl J Med 1987;317:65–69. 3587328 10.1056/NEJM198707093170201

[43] Massumi R. Familial Wolff-Parkinson-White syndrome with cardiomyopathy. Am J Med 1967;43:951–55. 4228766 10.1016/0002-9343(67)90254-9

[44] Gollob MH Green MS Tang AS. Identification of a gene responsible for familial Wolff–Parkinson–White syndrome. N Engl J Med 2001;344:1823–31. 11407343 10.1056/NEJM200106143442403

[45] Islam H Puttagunta SM Islam R. Risk of stroke with mitral stenosis: the underlying mechanism, treatment, and prevention. Cureus 2022;14:e23784. 35518523 10.7759/cureus.23784 PMC9063730

[46] MacRae C Ghaisas N Kass S. Familial hypertrophic cardiomyopathy with Wolff-Parkinson-White syndrome maps to a locus on chromosome 7q3. J Clin Investig 1995;96:1216–20. 7657794 10.1172/JCI118154 PMC185741

[47] Deal BJ Keane JF Gillette PC. Wolff-Parkinson-White syndrome and supraventricular tachycardia during infancy: management and follow-up. J Am Coll Cardiol 1985;5:130–35. 3964800 10.1016/s0735-1097(85)80095-4

[48] Khairy P Van Hare GF Balaji S. PACES/HRS expert consensus statement on the recognition and management of arrhythmias in adult congenital heart disease. Can J Cardiol 2014;30:e1–e63. 25262867 10.1016/j.cjca.2014.09.002

[49] Ghosh S Avari JN Rhee EK. Hypertrophic cardiomyopathy with preexcitation: insights from noninvasive electrocardiographic imaging (ECGI) and catheter mapping. J Cardiovasc Electrophysiol 2008;19:1215–17. 18479334 10.1111/j.1540-8167.2008.01203.x PMC2631093

[50] Murphy RT Mogensen J McGarry K. Adenosine monophosphate-activated protein kinase disease mimicks hypertrophic cardiomyopathy and Wolff-Parkinson-White syndrome: natural history. J Am Coll Cardiol 2005;45:922–30. 15766830 10.1016/j.jacc.2004.11.053

[51] Maron BJ. Clinical outcome and phenotypic expression in LAMP2 Cardiomyopathy. Jama 2009;301:1253–59. 19318653 10.1001/jama.2009.371 PMC4106257

[52] Koneru JN Wood MA Ellenbogen KA. Rare forms of preexcitation a case study and brief overview of familial forms of preexcitation. Circ Arrhythm Electrophysiol 2012;5:82–88. 10.1161/CIRCEP.111.968917 22895604

[53] Suzuki T Nakamura Y Yoshida S. Differentiating fasciculoventricular pathway from Wolff-Parkinson-White syndrome by electrocardiography. Heart Rhythm 2014;11:686–90. 24252285 10.1016/j.hrthm.2013.11.018

[54] Fitzsimmons PJ McWhirter PD Peterson DW. The natural history of Wolff-Parkinson-White syndrome in 228 military aviators: a long-term follow-up of 22 years. Am Heart J 2001;142:530–36. 11526369 10.1067/mhj.2001.117779

